# Mapping Divergent Subfield‐Specific Hippocampal Degeneration in Mild Cognitive Impairment Continuum: Volumetric, Cognitive, and Genetic Predictors of Accelerated Hippocampal Biological Aging

**DOI:** 10.1111/cns.70548

**Published:** 2025-07-29

**Authors:** Sadegh Ghaderi, Sana Mohammadi, Farzad Fatehi, for the Alzheimer's Disease Neuroimaging Initiative

**Affiliations:** ^1^ Department of Neuroscience and Addiction Studies, School of Advanced Technologies in Medicine Tehran University of Medical Sciences Tehran Iran; ^2^ Neuromuscular Research Center, Department of Neurology, Shariati Hospital Tehran University of Medical Sciences Tehran Iran

**Keywords:** hippocampal biological age, mild cognitive impairment (MCI), neurodegeneration, neuroimaging, structural MRI

## Abstract

**Objective:**

To investigate hippocampal subfield atrophy and biological aging across the mild cognitive impairment (MCI) continuum, we used data from the Alzheimer's Disease Neuroimaging Initiative (ADNI).

**Methods:**

A cohort of 49 participants, categorized as cognitively normal (CN, *n* = 16), early MCI (EMCI, *n* = 16), or late MCI (LMCI, *n* = 17), underwent comprehensive neuroimaging, neuropsychological, and genetic assessments. High‐resolution 3D T1‐weighted MRI scans were processed using the volBrain platform and hippocampal subfield segmentation (HIPS) pipeline to quantify hippocampal subfield volumes and estimate biological age. Statistical analyses, including ANCOVA and stepwise regression, were employed to evaluate group differences and identify predictors of hippocampal biological age.

**Results:**

The results revealed significant volumetric reductions in LMCI, particularly within the CA1, CA4/dentate gyrus (DG), and stratum radiatum/lacunosum/moleculare (SRLM) subfields, with pronounced lateralized effects. Clinical and demographic covariates attenuated group differences in biological age, but volumetric adjustments highlighted a significant distinction between EMCI and LMCI, with EMCI exhibiting a higher biological age. Cognitive performance, as measured by the Montreal Cognitive Assessment (MoCA), emerged as a consistent predictor of biological age, while APOE ε4 carrier status was significantly elevated in LMCI patients. Regression analyses identified divergent contributions of CA2/3 (positively associated) and CA4/DG (negatively associated) volumes to biological age, underscoring the subfield‐specific pathophysiological mechanisms. Asymmetry indices, although variably expressed across groups, offered limited predictive utility, with CA2/3 and CA4/DG asymmetries modestly influencing biological age.

**Conclusion:**

These findings support the integration of subfield‐specific hippocampal volumetry and cognitive assessments in early diagnostic frameworks while highlighting the need for longitudinal studies to elucidate causal pathways linking subfield atrophy, biological aging, and cognitive decline.

## Graphical abstract

Advanced hippocampal subfield volumetry reveals that distinct atrophy patterns predict accelerated biological aging and cognitive decline in mild cognitive impairment (MCI). These findings support subfield‐specific biomarkers for early Alzheimer's disease detection.

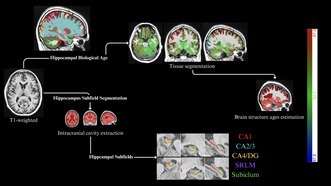

## 1 Introduction

The hippocampus is not a homogeneous structure; it is comprised of multiple subfields with distinct anatomies and functions. The progression of mild cognitive impairment (MCI) is associated with a pattern of hippocampal degeneration characterized by pronounced subfield‐specific atrophy [1, 2]. Recent advances in neuroimaging have underscored the hippocampus as a focal point of early AD pathology, with its subfields exhibiting distinct vulnerabilities to biological age. Although whole‐hippocampal volumetry has long been a cornerstone of AD research, growing evidence highlights the limitations of this approach, as heterogeneous subfield atrophy patterns may better predict cognitive decline and conversion to dementia [3].

Recent mechanistic studies have demonstrated that AD‐related neurofibrillary tau tangles first accumulate in the Cornu Ammonis 1 (CA1) before propagating to adjacent subfields, such as the subiculum and CA2/3 Alzheimer's [4, 5], whereas amyloid‐β pathology preferentially targets the CA4/dentate gyrus (DG) region and coincides with the early collapse of adult hippocampal neurogenesis in the DG [6]. This differential vulnerability underlies early atrophy patterns in MCI; however, transient synaptic and dendritic plasticity in CA2/3 may briefly mask volume loss before overt degeneration [7, 8]. In parallel, hemispheric asymmetries in subfield volumes have been linked to domain‐specific cognitive deficits, suggesting dynamic functional reorganization from a cognitively normal state to late MCI (LMCI) [9, 10].

Technological advancements in high‐resolution magnetic resonance imaging (MRI) and automated segmentation pipelines, such as the hippocampus subfield segmentation (HIPS) protocol [11], have revolutionized the precision of subfield volumetry. Concurrently, the concept of “biological brain age,” derived from machine learning models trained on neuroanatomical features, has emerged as a powerful biomarker to quantify deviations from normative aging trajectories and predict cognitive outcomes [12].

While prior studies have examined subfield atrophy, this is the first study to integrate volumetric subfield measures with a deep learning‐derived hippocampal biological age metric across the entire MCI continuum. This study addresses these gaps by investigating three aims: (1) characterizing subfield‐specific atrophy patterns across cognitively normal (CN), early MCI (EMCI), and LMCI groups using structural MRI (sMRI); (2) evaluating the predictive utility of the hippocampal biological age deep learning framework in distinguishing MCI stages; and (3) elucidating the roles of genetic phenotype, cognitive performance, and asymmetry indices in modulating subfield atrophy and biological aging.

## 2 Methods

### 2.1 Alzheimer's Disease Neuroimaging Initiative

The data used in this study were obtained from the ADNI database (https://ida.loni.usc.edu/). The ADNI was launched in 2003 as a public‐private partnership, led by Principal Investigator Michael W. Weiner, MD. The primary goal of ADNI is to test whether serial MRI, positron emission tomography (PET), other biological markers, and clinical and neuropsychological assessments can be combined to measure the progression of MCI and early AD. The participants were selected from the ADNI phases. The exclusion criteria were a history of stroke, major psychiatric disorder, severe head injury with loss of consciousness, uncontrolled hypertension or diabetes, and any MRI scan with motion or other artifacts preventing reliable subfield segmentation.

The diagnoses of EMCI and late LMCI for participants within the ADNI database were established by experienced clinicians based on comprehensive neurological and neuropsychological assessments. These diagnoses adhered to standardized ADNI diagnostic criteria, incorporating clinical evaluations and functional assessments. Further details on ADNI's diagnostic protocols are publicly available through the ADNI database resources.

### 2.2 MRI and Image Analysis

We used three‐dimensional (3D) accelerated sagittal T1‐weighted (T1‐w) images acquired using 3.0 Tesla MRI scanners equipped with phased‐array coils. Detailed MRI acquisition protocols are publicly accessible at https://adni.loni.usc.edu/data‐samples/adni‐data/neuroimaging/mri/mri‐scanner‐protocols/ and have been comprehensively described in previous publications [13, 14, 15]. The original MRI data were retrieved from the Laboratory of Neuro Imaging Image and Data Archive (https://ida.loni.usc.edu) on February 20, 2024, adhering to ADNI's data usage agreements.

For data conversion, the dcm2niix software, a part of the MRIcroGL suite, was employed to transform the T1‐w data from DICOM to NIfTI format, ensuring data anonymization. Subsequently, all T1‐w images underwent meticulous review to confirm isotropic structure and were standardized using FreeSurfer's mri_convert tool, resampling the data into 1 mm^3^ isotropic voxels.

Biological brain structural age (BSA) prediction [12] was conducted using the cloud‐based volBrain platform (https://volbrain.net/). This platform leverages deep learning models trained on a normative lifespan cohort spanning 0–100 years. It automates region‐specific age estimation by extracting anatomical features from T1‐w MRI scans and synthesizing them into global BSA metrics and BSA values for specific brain subregions, such as the bilateral hippocampus for each participant. For neuroanatomical segmentation, the AssemblyNet framework [16] was utilized alongside quality control procedures for image‐to‐template brain MRI affine registration [17] to facilitate the parcellation of subcortical regions, including the hippocampus.

The hippocampal subfield segmentation (HIPS) pipeline [11] is an advanced tool designed for the automatic segmentation of hippocampal subfields using either monomodal (T1‐w) or multimodal (T1‐w and T2‐w) MRI data. In addition to subfield segmentation, HIPS facilitates automated hippocampal segmentation and volumetric analysis. In this study, the “Winterburn” protocol was utilized [18], which divides the hippocampus into five distinct segments: CA1, CA2/CA3, CA4/DG, stratum radiatum/lacunosum/moleculare (SRLM), and subiculum. Furthermore, we extracted the Symmetry Index, which reports the percentage difference between the right and left volumes divided by their mean.

### 2.3 Neuropsychological and Genetic Tests

Neuropsychological evaluations encompassed the Clinical Dementia Rating (CDR) [19] scale, which serves as an index of functional ability, and the Mini‐Mental State Examination (MMSE) [20] and Montreal Cognitive Assessment (MoCA) [21] tests, which are global indices of cognitive function. Blood samples were collected from each participant to determine the apolipoprotein E (APOE) genotype. Participants were stratified into four APOE allele profiles: e4 (ε4ε4 or ε3ε4), e2 (ε2ε2 or ε2ε3), e3 (ε3ε3), and e24 (ε2ε4). Individuals possessing one or more ε4 alleles were designated APOE carriers (ε4+), whereas those lacking ε4 alleles were classified as non‐carriers (ε4−).

### 2.4 Statistical Analyses

Statistical analyses were conducted to evaluate group differences in the hippocampal subfield volume, biological age, and the influence of clinical, demographic, and genetic factors. Data distributions were assessed for normality using the Shapiro–Wilk test, which indicated nonnormal distributions (*p* < 0.05). Nonparametric tests were used for group comparisons.

One‐way ANOVA was used to compare the total hippocampal and subfield volumes across the three diagnostic groups: CN, EMCI, and LMCI. Post hoc Bonferroni tests were used to identify specific group differences. For non‐normally distributed data, the Kruskal–Wallis *H* test was used, followed by Mann–Whitney *U* tests for pairwise comparisons. Effect sizes were reported using partial eta‐squared (*η* ^2^) for ANOVA and Cohen's *d* for post hoc tests. Two ANCOVA models were constructed to assess group differences in hippocampal biological age, while controlling for covariates. The first model was adjusted for clinical and demographic covariates, including years of education, CDR, MMSE, MoCA, sex, and APOE carrier status. The second model incorporated volumetric measures of the hippocampal subfields as additional covariates. Estimated Marginal Means (EMMs) were calculated for each group, and Bonferroni corrections were applied for pairwise comparisons. Homogeneity of variance was confirmed using Levene's test.

Multiple regression analyses were performed to identify key predictors of hippocampal biological age. The initial pool of predictors included diagnostic group, sex, APOE status, years of education, CDR, MMSE, MoCA scores, and hippocampal subfield volumes. All predictor variables were evaluated for multicollinearity; pairwise correlations were < 0.60, and VIF values for all covariates in the ANCOVA and regression models were < 5, indicating acceptable levels of collinearity. Predictors were sequentially added or removed based on their contribution to the model, with a significance threshold of *p* < 0.05 for inclusion and *p* > 0.10 for exclusion. Predictors with *p*‐values falling between 0.05 and 0.10 were neither added nor retained in the final stepwise model.

Model fit was assessed using *R* ^2^ and adjusted *R* ^2^ values, and standardized beta coefficients (*β*) were used to indicate the strength and direction of the predictor effects. Asymmetry indices for the hippocampal subfields were calculated as the percentage difference between the right and left volumes, divided by their mean. Group differences in asymmetry indices were evaluated using ANCOVA, controlling for chronological age, TIV, years of education, and sex as relevant covariates. Forced‐entry and stepwise regression models were employed to assess the predictive utility of asymmetry indices for hippocampal biological age.

All analyses were performed using the SPSS software (version 27.0). A significance threshold of *p* < 0.05 was applied for all tests, with Bonferroni corrections used to adjust for multiple comparisons. The effect sizes were reported to provide a context for the magnitude of the observed differences.

## 3 Results

### 3.1 Sample Results

**FIGURE 1:**
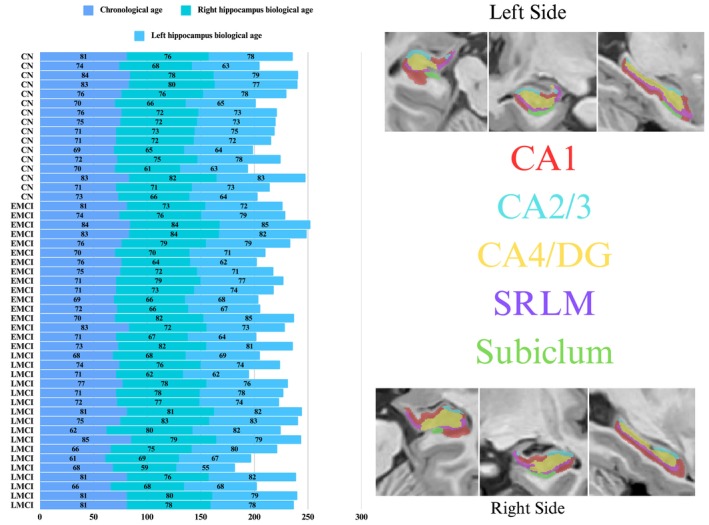
Chronological age, hippocampal biological age, and subfield volumes across diagnostic groups: The left side represents chronological age alongside biological age estimates of the left and right hippocampus, stratified by diagnostic group (CN, cognitively normal; EMCI, early mild cognitive impairment; LMCI, late mild cognitive impairment). Numerical values represent individual participant data, with rows corresponding to chronological age, right hippocampal biological age, and left hippocampal biological age. On the right side, subfield volumetric data (CA1, CA2/3, CA4/dentate gyrus [DG], stratum radiatum/lacunosum/moleculare [SRLM], and subiculum) for the left and right hemispheres are displayed.

**FIGURE 2:**
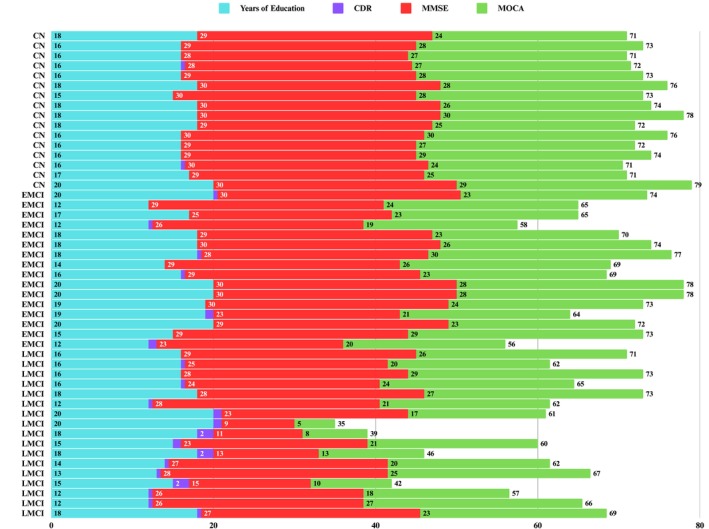
Education and clinical characteristics by the diagnostic group [Columns include years of education, Clinical Dementia Rating (CDR), Mini‐Mental State Examination (MMSE), and Montreal Cognitive Assessment (MoCA) scores. Rows are stratified by diagnostic group (CN, EMCI, LMCI)].

**FIGURE 3:**
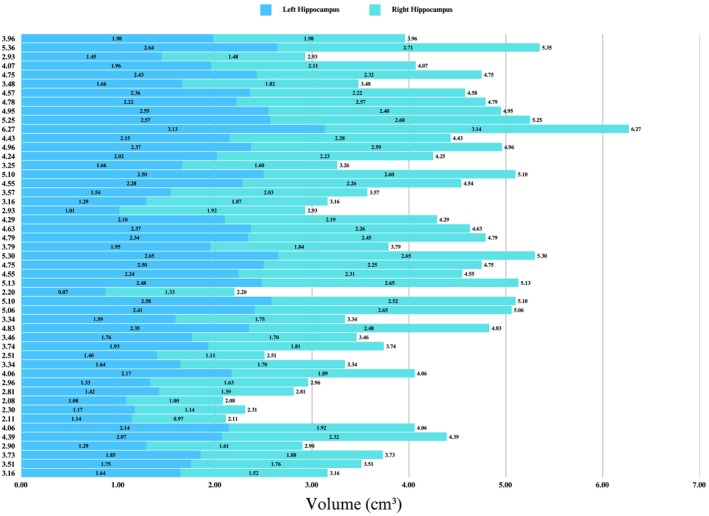
Left and right hippocampal subfield volumes. Volumetric data (cm^3^) for the left and right hippocampal subfields (CA1, CA2/3, CA4/DG, SRLM, subiculum). Each row corresponds to a participant, with the stack total representing the overall volume.

**FIGURE 4:**
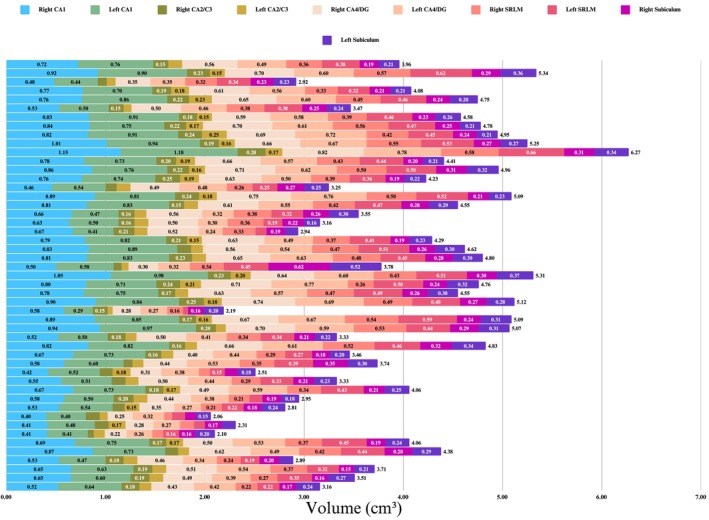
Hippocampal subfield volumetric comparisons by hemisphere and diagnostic group. The stacked row chart compares mean hippocampal subfield volumes (cm^3^) between left and right hemispheres across diagnostic groups (CN, EMCI, LMCI). Subfields include CA1, CA2/3, CA4/DG, SRLM, and subiculum. Each row corresponds to a participant, with the stack total representing the overall volume.

Baseline demographic, neuropsychological, clinical, and APOE data were collected and analyzed for 49 participants categorized as CN (*n* = 16), EMCI (*n* = 16), and LMCI (*n* = 17) (Table 1). Figure 1 shows the chronological age, bilateral hippocampal biological age, and subfield volume representations across diagnostic groups. No significant differences were observed in chronological age, total intracranial volume (TIV), years of education, handedness, or sex distribution (all *p* > 0.05). Summaries of the groups' education and clinical characteristics are reported (Figure 2). The total hippocampal volume (Figure 3) and subfield results (Figure 4), including CA1, CA2/3, CA4/DG, SRLM, and asymmetry indices, varied by group and are reported in Table 2.

**TABLE 1:** Demographics and clinical characteristics of cognitively normal (CN), early mild cognitive impairment (EMCI), and late MCI (LMCI).

	CN (*n* = 16)	EMCI (*n* = 16)	LMCI (*n* = 17)	*p*
Chronological age (year)^a^	74.94 (5.13)	74.94 (5.13)	72.94 (7.26)	0.586
Total intracranial volume (cm^3^)^a^	1400.60 (135.45)	1426.01 (136.34)	1370.78 (124.35)	0.656
Year of education^a^	17 (1)	17 (3)	16 (3)	0.341
Clinical dementia rating (CDR)^a^	0.1 (0.2)	0.3 (0.4)	0.8 (0.7)	< 0.001
Total MMSE score^a^	29 (1)	28 (2)	23 (7)	< 0.001
Total MoCA score^a^	27 (2)	24 (3)	20 (7)	< 0.001
Handedness, right (*n*/%)	16 (100.0%)	13 (81.3%)	16 (94.1%)	0.146
Sex, female (*n*/%)	10 (62.5%)	8 (50.0%)	13 (76.5%)	0.301
APOE, (+/%)	2 (12.5%)	3 (18.8%)	11 (64.7%)	0.001

^a^ Mean (SD, standard deviation).

**TABLE 2:** Bilateral hippocampal biological structure age, volume of the hippocampus, and hippocampal subfields of cognitively normal (CN), early mild cognitive impairment (EMCI), and late MCI (LMCI).

Measure	Area/index	CN		EMCI		LMCI	
		Mean	SD	Mean	SD	Mean	SD
Biological age (hippocampal structure age)	Right	72.06	5.77	74.31	6.72	74.66	6.85
	Left	72.44	6.52	74.40	6.94	74.62	7.96
Hippocampus volume (cm^3^)	Total	4.52	0.85	4.20	0.92	3.29	0.80
	Right	2.30	0.43	2.18	0.37	1.64	0.42
	Left	2.23	0.43	2.01	0.57	1.65	0.39
	Asymmetry^a^	3.07	6.21	11.41	20.11	−1.69	12.00
Cornu Ammonis 1 (CA1) volume (cm^3^)	Total	1.56	0.36	1.46	0.35	1.18	0.26
	Right	0.79	0.18	0.76	0.15	0.59	0.14
	Left	0.78	0.18	0.70	0.21	0.59	0.13
	Asymmetry	1.27	9.41	11.01	22.02	−1.27	11.52
Cornu Ammonis 2 and 3 (CA2 and CA3) volume (cm^3^)	Total	0.37	0.09	0.32	0.08	0.29	0.05
	Right	0.20	0.05	0.18	0.04	0.15	0.04
	Left	0.16	0.04	0.13	0.04	0.14	0.03
	Asymmetry	16.97	21.28	31.80	25.45	8.66	30.51
Cornu Ammonis 4 and dentate gyrus (CA4 and DG) volume (cm^3^)	Total	1.21	0.22	1.07	0.29	0.86	0.22
	Right	0.63	0.11	0.58	0.13	0.43	0.12
	Left	0.58	0.11	0.50	0.17	0.42	0.11
	Asymmetry	7.80	7.68	16.82	23.14	1.67	17.51
Stratum radiatum, lacunosum and moleculare (SRLM) volume (cm^3^)	Total	0.88	0.20	0.79	0.24	0.55	0.24
	Right	0.44	0.10	0.40	0.10	0.27	0.12
	Left	0.45	0.11	0.39	0.15	0.28	0.13
	Asymmetry	−1.81	8.25	9.82	44.07	−5.12	23.50
Subiculum volume (cm^3^)	Total	0.50	0.08	0.55	0.18	0.42	0.11
	Right	0.24	0.04	0.27	0.10	0.20	0.06
	Left	0.25	0.05	0.28	0.09	0.22	0.06
	Asymmetry	−3.56	10.00	−5.96	20.21	−12.33	22.80

^a^ Asymmetry Index: percentage difference between the right and left volumes divided by their mean.

Table 3 presents a detailed table presenting the results of two ANCOVA models and a stepwise regression analysis for the biological age of the left and right hippocampi. The ANCOVA models included one adjusted for clinical and demographic covariates and another adjusted for volumetric covariates across the CN, EMCI, and LMCI groups. Stepwise regression was used to identify the key predictors of hippocampal biological age. Additionally, EMMs adjusted for asymmetry indices are provided in a note for completeness. This table was designed for academic purposes to ensure clarity and rigor in presenting the statistical outcomes.

**TABLE 3:** ANCOVA and regression results for left and right hippocampal biological age.

Analysis	Left hippocampus biological age	Right hippocampus biological age
ANCOVA model 1: clinical and demographic covariates		
Overall model	*F* (15, 33) = 2.014 *p* = 0.046 *η* ^2^ = 0.478	*F* (15, 33) = 1.596 *p* = 0.129 *η* ^2^ = 0.420
Group effect	*F* (2, 33) = 0.764 *p* = 0.474 *η* ^2^ = 0.044	*F* (2, 33) = 0.502 *p* = 0.610 *η* ^2^ = 0.029
Significant covariates	MOCA: *F* (1, 33) = 4.651 *p* = 0.038 *η* ^2^ = 0.124	MOCA: *F* (1, 33) = 4.441 *p* = 0.043 *η* ^2^ = 0.119
Estimated marginal means (SE, 95% CI)	CN: 73.93 (2.55, 68.74–79.11) EMCI: 75.67 (2.13, 71.33–80.01) LMCI: 72.10 (1.99, 68.04–76.15)	CN: 73.59 (2.44, 68.63–78.55) EMCI: 75.41 (2.04, 71.27–79.56) LMCI: 72.69 (1.90, 68.82–76.57)
Pairwise comparisons	No significant differences	No significant differences
ANCOVA model 2: volumetric covariates		
Overall model	*F* (17, 31) = 5.450 *p* < 0.001 *η* ^2^ = 0.749	*F* (17, 31) = 3.860 *p* = 0.001 *η* ^2^ = 0.679
Group effect	*F* (2, 31) = 5.285 *p* = 0.011 *η* ^2^ = 0.254	*F* (2, 31) = 5.191, *p* = 0.011, *η* ^2^ = 0.251
Significant covariates	None individually significant	None individually significant
Estimated marginal means (SE, 95% CI)	CN: 73.12 (1.83, 69.39–76.84) EMCI: 77.87 (1.58, 74.65–81.08) LMCI: 70.66 (1.47, 67.66–73.66)	CN: 73.77 (2.03, 69.62–77.91) EMCI: 77.65 (1.57, 74.45–80.84) LMCI: 70.45 (1.50, 67.39–73.51)
Pairwise comparisons	EMCI > LMCI (mean diff = 7.21, *p* = 0.009)	EMCI > LMCI (mean diff = 7.20, *p* = 0.009)
Stepwise regression		
Final model	*R* ^2^ = 0.603 adj *R* ^2^ = 0.576 *F* (3, 45) = 22.753 *p* < 0.001	*R* ^2^ = 0.421 adj *R* ^2^ = 0.395 *F* (2, 46) = 16.700 *p* < 0.001
Significant predictors	Left CA2/CA3 volume: *B* = 98.25, SE = 20.59, *β* = 0.549, *t* = 4.772, *p* < 0.001 Left CA4/DG volume: *B* = −47.32, SE = 5.73, *β* = −0.974, *t* = −8.252, *p* < 0.001 Education: *B* = 0.64, SE = 0.28, *β* = 0.220, *t* = 2.262, *p* = 0.029	Right SRLM volume: *B* = −37.79, SE = 6.63, *β* = −0.755, *t* = −5.698, *p* < 0.001 Right CA2/CA3 volume: *B* = 38.98, SE = 17.68, *β* = 0.292, *t* = 2.205, *p* = 0.033

### 3.2 Hippocampal Volume and Subfield Comparisons

One‐way ANOVA revealed significant group differences in total hippocampal volume (*F* (2, 46) = 9.17, *p* < 0.001, *η* ^2^ = 0.285), with post hoc Bonferroni tests showing that LMCI had smaller volumes than both CN (MD, mean difference = 1.23 cm^3^, *p* < 0.001) and EMCI (MD = 0.91 cm^3^, *p* = 0.012), while CN and EMCI did not differ (*p* = 0.866). A similar pattern emerged for right hippocampal volume, where the LMCI participants also exhibited significantly lower volumes than both CN (MD = 0.66 cm^3^, *p* < 0.001) and EMCI individuals (MD = 0.55 cm^3^, *p* = 0.001). The hippocampal subfields showed progressive atrophy across the MCI stages. Total CA1 volume differed significantly (*F* (2, 46) = 6.30, *p* = 0.004, *η* ^2^ = 0.215), driven by LMCI deficits relative to CN (MD = 0.39 cm^3^, *p* = 0.004) and EMCI (MD = 0.28 cm^3^, *p* = 0.048). Lateralized effects emerged in the right CA1 (*p* = 0.001) and left CA1 (*p* = 0.015), with LMCI < CN, bilaterally. The total CA2/3 volume decreased across groups (*F* (2, 46) = 5.04, *p* = 0.011, *η* ^2^ = 0.180), particularly between CN and LMCI (MD = 0.08 cm^3^, *p* = 0.008). Right CA2/3 showed a stronger group effect (*F* (2, 46) = 4.77, *p* = 0.013) than left (*F* (2, 46) = 3.28, *p* = 0.047). Marked CA4/DG atrophy occurred in LMCI (*F* (2, 46) = 8.87, *p* = 0.001, *η* ^2^ = 0.278), with LMCI < CN (MD = 0.36 cm^3^, *p* < 0.001) and LMCI < EMCI (MD = 0.21 cm^3^, *p* = 0.048). The left CA4/DG group showed similar effects (*p* = 0.005). Total SRLM volume differed substantially (*F* (2, 46) = 9.90, *p* < 0.001, *η* ^2^ = 0.301), with LMCI < CN (MD = 0.34 cm^3^, *p* < 0.001) and LMCI < EMCI (MD = 0.25 cm^3^, *p* = 0.009). The right SRLM showed a greater effect size (*F* (2, 46) = 11.41, *p* < 0.001, *η* ^2^ = 0.332) than the left SRLM.

Significant differences emerged for left hippocampal volume (*H* = 11.39, *p* = 0.003), right CA4/DG (*H* = 17.24, *p* < 0.001), left SRLM (*H* = 11.98, *p* = 0.003), and both total and right subiculum volumes (*H* values ranging from 7.67 to 10.53, all *p* < 0.05). Follow‐up Mann–Whitney *U* tests clarified these group comparisons; differences between the CN and LMCI groups were significant across several regions (e.g., left hippocampus *U* = 41.0, *p* = 0.001; right CA4/DG *U* = 31.5, *p* < 0.001), and when comparing the EMCI and LMCI groups, significant differences were also observed (e.g., left hippocampus *U* = 77.5, *p* = 0.035; right CA4/DG *U* = 48.5, *p* = 0.002). In contrast, comparisons between the CN and EMCI groups generally did not reach statistical significance, suggesting that the volumetric reduction was most pronounced in the LMCI stage.

### 3.3 Left Hippocampal Biological Age

#### 3.3.1 Adjustment for Clinical and Demographic Covariates

The biological age of the left hippocampus served as the dependent variable, with groups (CN, EMCI, and LMCI) as the between‐subjects factor. Covariates included education, CDR, MMSE, and MoCA, along with sex and APOE carrier and non‐carrier categories, as additional factors, including their interactions with groups. The overall model was statistically significant (*F* (15, 33) = 2.014, *p* = 0.046, partial *η* ^2^ = 0.478), explaining 47.8% of the variance (adjusted *R* ^2^ = 0.241). However, the main effect of group was not significant (*F* (2, 33) = 0.764, *p* = 0.474, partial *η* ^2^ = 0.044), indicating no substantial differences in left hippocampal biological age across groups after covariate adjustment. Among the covariates, only MoCA emerged as a significant predictor (*F* (1, 33) = 4.651, *p* = 0.038, partial *η* ^2^ = 0.124), with a negative relationship (*B* = −0.900, standard error [SE] = 0.417, *t* = −2.157, *p* = 0.038), suggesting that higher MoCA scores (indicative of better cognitive performance) were associated with younger hippocampal biological age. Sex, APOE status, and their interactions with the group did not reach significance (all *p* > 0.05). The EMMs adjusted for covariates were CN = 73.925 (SE = 2.551), EMCI = 75.669 (SE = 2.132), and LMCI = 72.096 (SE = 1.992). Bonferroni‐corrected pairwise comparisons revealed no significant group differences (e.g., EMCI vs. LMCI: mean difference = 3.573, *p* = 0.675; CN vs. EMCI: mean difference = −1.744, *p* = 1.000). Levene's test confirmed the homogeneity of variance (*F* (11, 37) = 1.146, *p* = 0.356), supporting the model's assumptions.

#### 3.3.2 Adjustment for Volumetric Covariates

The second ANCOVA incorporated volumetric measures of the left hippocampus and its subfields as covariates, along with group, sex, APOE status, and their interactions. This model was highly significant (*F* (17, 31) = 5.450, *p* < 0.001, partial *η* ^2^ = 0.749), accounting for 74.9% of the variance (adjusted *R* ^2^ = 0.612). Unlike the first model, the main effect of group was significant (*F* (2, 31) = 5.285, *p* = 0.011, partial *η* ^2^ = 0.254), indicating detectable group differences in the biological age of the left hippocampus when the volumetric measures were controlled. Bonferroni‐adjusted pairwise comparisons revealed a significant difference between EMCI and LMCI (mean difference = 7.205, SE = 2.247, *p* = 0.009), with EMCI exhibiting a higher biological age (77.865, SE = 1.578) than LMCI (70.659, SE = 1.470). The differences between CN (73.115, SE = 1.825) and EMCI (mean difference = −4.750, *p* = 0.179) and between CN and LMCI (mean difference = 2.455, *p* = 0.960) were not significant. Levene's test supported equal variance (*F* (11, 37) = 1.729, *p* = 0.105). None of the volumetric covariates individually reached significance (*p* > 0.05), although the sex × APOE carrier interaction approached significance (*F* (1, 31) = 4.023, *p* = 0.054, partial *η* ^2^ = 0.115). Parameter estimates highlighted a significant effect for the EMCI group (*B* = 12.977, SE = 5.262, *t* = 2.466, *p* = 0.019) relative to LMCI, reinforcing the group distinction.

#### 3.3.3 Regression Results

A subsequent stepwise multiple regression analysis was performed to determine the clinical and neuroanatomical predictors that best accounted for the variance in the biological age of the left hippocampus. The initial pool of predictors included group, sex, APOE status, years of education, CDR, MMSE, MoCA scores, and the same set of left hippocampal subregion volumes. The final regression model, which emerged after the stepwise selection procedure, included Left CA2 and CA3 volumes, Left CA4 and DG volumes, and years of education. This model explained 60.3% of the variance in the biological age of the left hippocampus (*R* ^2^ = 0.603, Adjusted *R* ^2^ = 0.576; *F* (3, 45) = 22.753, *p* < 0.001). Specifically, greater volumes in the left CA2 and CA3 regions were associated with an increase in biological age (*B* = 98.249, *t* = 4.772, *p* < 0.001), whereas an increased left CA4/DG volume predicted a lower biological age (*B* = −47.323, *t* = −8.252, *p* < 0.001). In addition, higher years of education were significantly associated with higher left hippocampal biological age (*B* = 0.641, *t* = 2.262, *p* = 0.029).

### 3.4 Right Hippocampal Biological Age

#### 3.4.1 Adjustment for Clinical and Demographic Covariates

As with the left side, initial adjustments were performed for the biological age of the right hippocampus. The overall model did not achieve statistical significance (*F* (15, 33) = 1.596, *p* = 0.129, partial *η* ^2^ = 0.420), accounting for 42.0% of the variance (adjusted *R* ^2^ = 0.157). The main effect of group was also not significant (*F* (2, 33) = 0.502, *p* = 0.610, partial *η* ^2^ = 0.029), indicating no notable differences in the biological age of the right hippocampus across groups when adjusted for these covariates. Among the covariates, only MoCA emerged as a significant predictor (*F* (1, 33) = 4.441, *p* = 0.043, partial *η* ^2^ = 0.119), showing a negative association with the biological age of the right hippocampus (*B* = −0.840, SE = 0.399, *t* = −2.107, *p* = 0.043). Other covariates, including education, CDR, MMSE score, sex, APOE status, and their interactions with group, were not significant (*p* > 0.05). The EMMs adjusted for covariates were CN = 73.594 (SE = 2.438), EMCI = 75.412 (SE = 2.037), and LMCI = 72.691 (SE = 1.904). Bonferroni‐corrected pairwise comparisons revealed no significant group differences (e.g., CN vs. EMCI: mean difference = −1.819, *p* = 1.000; EMCI vs. LMCI: mean difference = 2.721, *p* = 0.995). Levene's test confirmed the homogeneity of the variance (*F* (11, 37) = 1.667, *p* = 0.120), supporting the model's assumptions.

#### 3.4.2 Adjustment for Volumetric Covariates

The second ANCOVA incorporated volumetric measures and accounted for a second adjustment. This model demonstrated high statistical significance (*F* (17, 31) = 3.860, *p* = 0.001, partial *η* ^2^ = 0.679), explaining 67.9% of the variance (adjusted *R* ^2^ = 0.503). A significant main effect of group was observed (*F* (2, 31) = 5.191, *p* = 0.011, partial *η* ^2^ = 0.251), indicating notable group differences in the biological age of the right hippocampus when the structural brain measures were controlled. Bonferroni‐adjusted pairwise comparisons revealed a significant difference between the EMCI and LMCI groups (mean difference = 7.195, SE = 2.244, *p* = 0.009), with the EMCI group exhibiting a higher biological age (77.645, SE = 1.568) than the LMCI group (70.450, SE = 1.501). However, the differences between the CN (73.767, SE = 2.033) and EMCI groups (mean difference = −3.879, *p* = 0.395) and between the CN and LMCI groups (mean difference = 3.316, *p* = 0.650) were not statistically significant. Levene's test confirmed the assumption of equal variance (*F* (11, 37) = 1.341, *p* = 0.242). None of the individual volumetric covariates were statistically significant (*p* > 0.05). Nevertheless, the interaction between group and APOE carrier status approached significance (*F* (2, 31) = 1.288, *p* = 0.290, partial *η* ^2^ = 0.077). Parameter estimates suggested a marginally significant effect for the EMCI group relative to the LMCI group (*B* = 9.510, SE = 5.204, *t* = 1.828, *p* = 0.077).

#### 3.4.3 Regression Results

Stepwise multiple regression analysis was performed with the biological age of the right hippocampus as the dependent variable and an array of predictors (similar to those used for the left side). The final regression model included only two volumetric predictors: the right SRLM volume and the right CA2/3 volume. This model accounted for 42.1% of the variance (*R* ^2^ = 0.421, adjusted *R* ^2^ = 0.395). Specifically, a lower volume in the right SRLM was significantly associated with a higher biological age in the right hippocampus (*B* = −37.79, SE = 6.63, *t* = −5.70, *p* < 0.001, *β* = −0.755), whereas a higher volume in the right CA2/3 subregion predicted a higher biological age in the right hippocampus (*B* = 38.98, SE = 17.68, *t* = 2.21, *p* = 0.033, *β* = 0.292).

### 3.5 Patterns of Asymmetry and as Predictors of Hippocampal Biological Age

**FIGURE 5:**
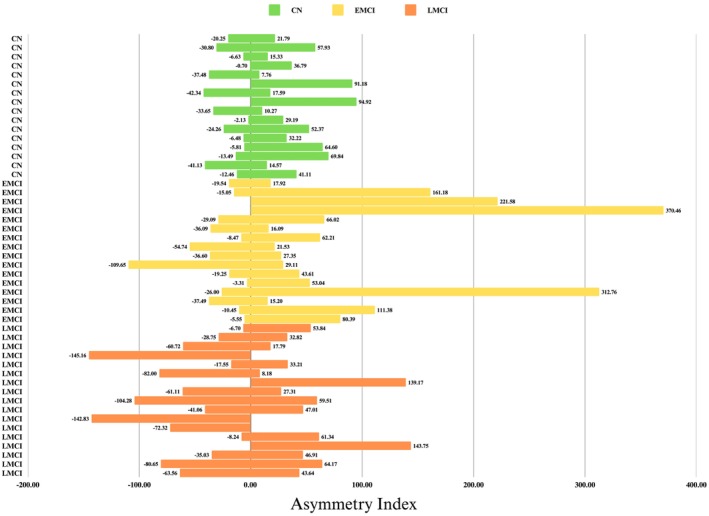
Hippocampal subfield asymmetry indices. The asymmetry indices (%), calculated as [(Right Volume − Left Volume)/Total Volume] × 100, for hippocampal subfields (CA1, CA2/3, CA4/DG, SRLM, and subiculum). Positive values represent rightward asymmetry, while negative values represent leftward asymmetry.

The asymmetry indices varied according to subfield and group (Figure 5). EMCI exhibited pronounced rightward asymmetry in the total hippocampus (11.41% ± 20.11%) and CA1 (11.01% ± 22.02%), whereas LMCI showed leftward asymmetry in the total hippocampus (−1.69% ± 12.00%) and subiculum (−12.33% ± 22.80%). CA2/3 asymmetry was maximal in EMCI (31.80% ± 25.45%), exceeding both CN (16.97% ± 21.28%) and LMCI (8.66% ± 30.51%; *p* = 0.013 for EMCI vs. LMCI).

A series of ANCOVAs assessed group differences in the left and right hippocampal biological age while controlling for asymmetry indices across hippocampal subfields. For left hippocampal biological age, the overall model was not significant (*F* (8, 40) = 1.52, *p* = 0.181, *η* ^2^ = 0.23), with no group differences observed between CN, EMCI, and LMCI (*F* (2, 40) = 0.10, *p* = 0.903, *η* ^2^ = 0.005). However, asymmetry in CA2/3 emerged as a significant covariate (*F* (1, 40) = 4.50, *p* = 0.04, *η* ^2^ = 0.101), where greater rightward asymmetry in CA2/3 predicted lower left hippocampal biological age (*B* = −0.21, *p* = 0.04). No other asymmetry indices contributed significantly to the model. The ANCOVA model was also not significant for the right hippocampal biological age (*F* (8, 40) = 1.61, *p* = 0.154, *η* ^2^ = 0.24), with no group effects (*F* (2, 40) = 0.29, *p* = 0.753, *η* ^2^ = 0.014). No other asymmetry indices contributed significantly to the model. Pairwise comparisons of the adjusted marginal means revealed no significant group differences after the Bonferroni correction (all *p* > 0.05).

Forced‐entry regression and stepwise models were used to evaluate whether the hippocampal asymmetry indices predicted the biological age of the left and right hippocampi. For left hippocampal biological age, the full model (including all asymmetry indices and groups) explained 23.2% of the variance (*R* ^2^ = 0.232, *F* (7, 41) = 1.77, *p* = 0.119), but the difference was not significant. Only CA2/3 asymmetry independently predicted left biological age (*B* = −0.21), with greater rightward CA2/3 asymmetry associated with younger biological age. For the right hippocampal biological age, stepwise regression identified CA4/DG asymmetry as a predictor accounting for 8.2% of the variance (*R* ^2^ = 0.082, *F* (1, 47) = 4.18, *p* = 0.046). Greater rightward CA4/DG asymmetry predicted an older right hippocampal biological age (*B* = 0.10, *p* = 0.046). No other asymmetry indices or group memberships were included in the model.

After adjusting for hippocampal subfield asymmetry indices, the EMMs for hippocampal biological age revealed modest upward trends across diagnostic groups, although there was considerable overlap in the 95% confidence intervals. For the left hippocampus, the EMMs were 73.19 (SE = 1.736) for CN subjects, 74.08 (SE = 1.837) for individuals with EMCI, and 74.21 (SE = 1.788) for those with LMCI, with the 95% CIs ranging from 69.68 to 76.70, 70.37 to 77.80, and 70.60 to 77.82, respectively. Similarly, for the right hippocampus, the EMMs were 72.75 (SE = 1.564) for CN, 73.89 (SE = 1.655) for EMCI, and 74.41 (SE = 1.612) for LMCI, with corresponding confidence intervals of 69.59–75.92, 70.54–77.23, and 71.15–77.66. Although there is a subtle trend toward increased biological age from CN through LMCI, the overlapping confidence intervals and non‐significant ANCOVA group effects suggest that when hippocampal asymmetry indices are considered, these differences are not robust enough to reliably distinguish diagnostic groups.

## 4 Disscusion

### 4.1 Summary of Key Findings

In summary, this study yielded three principal findings: (1) pronounced CA1, CA4/DG, and SRLM atrophy in LMCI versus CN and EMCI; (2) MoCA performance and APOE ε4 status as robust predictors of hippocampal biological age; and (3) the limited predictive value of subfield asymmetry indices. Below, we elaborate on their mechanistic and clinical significance.

### 4.2 Hippocampal Subfield Vulnerability Reflects Progressive Neurodegeneration

The results indicate significant group differences in total hippocampal volume, with LMCI participants exhibiting smaller volumes compared to both CN and EMCI groups, where hippocampal subfields are differentially affected by hippocampal atrophy in MCI [22, 23, 24, 25]. These findings suggest that the rate of hippocampal decline and the extent of volume loss occur on a continuum from normal aging to MCI to AD [26, 27, 28, 29, 30, 31]. These results are consistent with other studies showing that hippocampal subfields are not equally affected by normal aging, MCI, and AD and that their atrophy is selectively associated with declines in specific cognitive domains [27, 32, 33, 34, 35, 36]. Hippocampal subfield alterations, particularly within the CA1, CA4/DG, and SRLM subfields, may be highly susceptible to neurofibrillary tangle accumulation [37, 38, 39, 40], amyloid‐β deposition [41, 42], synaptic loss [43], pattern separation deficits [44, 45, 46, 47], impaired neurogenesis [48, 49], early volumetric reductions, and cortical disconnection [39, 50, 51, 52, 53], and involvement in spatial and contextual memory circuit disruption [54, 55, 56].

### 4.3 Genetic Risk and Cognitive Predictors of Biological Age

The higher APOE ε4 carrier prevalence in LMCI (64.7%) compared to CN (12.5%) and EMCI (18.8%) supports genetic vulnerability, consistent with studies linking APOE ε4 to increased AD risk in genomic studies across the lifespan [57, 58, 59]. APOE ε4 carriers exhibit heightened susceptibility to amyloid‐β aggregation [60] and synaptic dysfunction [61], particularly within the medial temporal lobe. However, the lack of a significant APOE × group interaction in our models suggests that ε's effects may be mediated through downstream neurodegenerative pathways, rather than by direct volumetric influences. This aligns with meta‐analyses showing that APOE ε4 modulates AD risk through interactions with amyloid pathology rather than through isolated structural changes [62].

### 4.4 Hemispheric Asymmetry

The lateralized volumetric declines observed in this study echo prior reports of asymmetric hippocampal degeneration in early AD [1, 63, 64]. While the left hippocampus is traditionally associated with verbal memory, the right hippocampus plays a dominant role in visuospatial processing, which may deteriorate earlier in MCI [65, 66]. However, the biological significance of lateralized atrophy remains unclear. Some studies have proposed that right hippocampal volume loss predicts conversion to AD more robustly than left‐sided changes [67], while others attribute asymmetry to methodological variability in segmentation protocols [68]. Our findings advocate for hemisphere‐specific analyses in future studies to clarify these discrepancies.

### 4.5 Brain Maintenance Hypothesis and Age–Atrophy Paradox

Biological age models have revealed a nuanced relationship among hippocampal aging, volumetric measures, and cognitive performance. While clinical covariates attenuated group differences in biological age, the inclusion of volumetric measures unmasked a significant distinction between EMCI and LMCI, with EMCI exhibiting paradoxically higher biological age. This finding suggests that structural atrophy may mediate accelerated biological aging trajectories in later MCI stages, consistent with the “brain maintenance” hypothesis, which posits that preserved neuroanatomical integrity buffers cognitive decline [69, 70]. Furthermore, this could indicate early structural changes in EMCI, such as altered shape or texture, which are captured by the volBrain model, but not by volume measures alone. In LMCI, severe atrophy might disrupt the ability of the model to accurately estimate age, as it was trained on cohorts with a predominantly lower mean age of [12]. This suggests that biological age may be a sensitive marker for EMCI, potentially aiding early detection; however, its interpretation in later stages requires caution.

MoCA Scores Inversely Correlate with Hippocampal Biological Age. The association between MoCA scores and lower hippocampal biological age underscores the value of cognitive assessments in monitoring neurodegeneration in MCI [71]. This finding aligns with evidence that cognitive decline correlates with structural brain changes that are predictive of MCI outcomes [72]. The inverse relationship between MoCA scores and biological age is consistent with longitudinal studies linking poor cognitive performance to accelerated brain aging, further emphasizing the sensitivity of MoCA to early neurodegenerative changes [21, 73]. Nevertheless, the contrasting contributions of CA2/3 (positive association) and CA4/DG (negative association) volumes to biological age require further exploration. The unexpected positive correlation between CA2/3 volume and biological age challenges conventional expectations, as larger volumes are typically indicative of healthier brain tissue. This discrepancy may reflect compensatory mechanisms such as transient synaptic expansion, glial hypertrophy, or early pathological processes preceding significant atrophy [8, 74]. In contrast, the protective role of CA4/DG volume aligns with its established involvement in neurogenesis and resilience against tau pathology [75].

### 4.6 Subfield Asymmetry Indices Provide Limited Predictive Utility

Although variably expressed across groups, the asymmetry indices demonstrated limited utility in predicting biological age. The rightward CA2/3 asymmetry in the EMCI and leftward subiculum asymmetry in the LMCI may reflect dynamic reorganization processes during MCI progression [76, 77, 78]. For instance, rightward hippocampal asymmetry may be linked to compensatory recruitment in early AD, whereas leftward shifts may signify maladaptive plasticity in the advanced stages [9]. However, the modest effect sizes of the asymmetry indices in our regression models suggest that interhemispheric volumetric differences alone are insufficient biomarkers for age estimation. This contrasts with studies emphasizing the prognostic value of asymmetry in AD [76], highlighting the need for shape asymmetry analysis [79] or multimodal approaches integrating machine learning with other advanced MRI methods or tau‐PET to disentangle clinical relevance [80, 81].

### 4.7 Methodological and Future Considerations

This study has several limitations. The study's cross‐sectional design and limited sample size underscore the importance of rigorous case–control matching of key demographic and anatomical variables, specifically age, sex, years of education, handedness, and TIV. This approach minimizes confounding factors and ensures comparability across CN, EMCI, and LMCI groups. However, strict matching criteria inherently constrain recruitment numbers and reduce the statistical power to detect subtle, subfield‐specific effects. Longitudinal studies tracking the subfield changes from EMCI to LMCI are required to establish temporality. Larger cohorts could help to validate these findings. Future research should incorporate AD‐specific neuroimaging biomarkers (e.g., amyloid‐PET), as well as blood and CSF biomarkers, to refine these models.

## 5 Conclusions

These findings underscore a progressive pattern of hippocampal degeneration across the MCI continuum, characterized by pronounced subfield‐specific atrophy in the LMCI, particularly within the CA1, CA4/DG, and SRLM regions, along with lateralized volumetric declines. While clinical and demographic covariates attenuated group differences in hippocampal biological age, controlling for volumetric measures revealed a significant distinction between EMCI and LMCI, suggesting that structural atrophy mediates biological aging trajectories. MoCA has emerged as a consistent predictor of biological age, aligning with its role as a marker of neurodegeneration. Regression analyses highlighted the divergent contributions of CA2/3 (positively associated) and CA4/DG (negatively associated) volumes to biological age, implicating subfield‐specific pathophysiological mechanisms. Although variably expressed across groups, asymmetry indices offered limited predictive utility, with CA2/3 and CA4/DG asymmetries modestly influencing the biological age. The elevated prevalence of APOE ε4 carriers in LMCI further emphasizes genetic vulnerability in later stages of MCI. These results support the integration of subfield‐specific hippocampal volumetry and cognitive assessments in early diagnostic frameworks while underscoring the need for longitudinal studies to elucidate causal pathways linking subfield atrophy, biological aging, and cognitive decline.

## Conflicts of Interest

The authors declare no conflicts of interest.

## Data Availability

The data that support the findings of this study are available from the corresponding author upon reasonable request.
